# Mothers' responses to relational savoring as a function of attachment: A qualitative study

**DOI:** 10.1002/imhj.70096

**Published:** 2026-05-15

**Authors:** Matthew J. Marvin, Patricia A. Smiley, Breana R. Cervantes, Margaret L. Kerr, Jessica L. Borelli

**Affiliations:** ^1^ Michigan State University East Lansing Michigan USA; ^2^ Pomona College Claremont California USA; ^3^ University of Houston Houston Texas USA; ^4^ University of Wisconsin–Madison Madison Wisconsin USA; ^5^ University of California Irvine Irvine California USA

**Keywords:** attachment, parent‐child relationships, qualitative, relational savoring

## Abstract

A person's state of mind with respect to attachment, measured by the Adult Attachment Interview (AAI), represents how the individual perceives, accesses, and processes attachment‐related content. One's state of mind with respect to attachment is thought to guide behavior in relationships, including caregiving relationships, and thus may have important implications for parents’ engagement in parenting interventions. *Relational savoring* (RS) is a brief attachment‐based intervention that has been shown to increase mothers’ parenting sensitivity, feelings of closeness to their child, and positive emotion. However, it remains unknown how mothers with different attachment profiles respond to this intervention. In the current illustrative multiple case study, we examine how three mothers (*M_age_ = *31.0 years, *SD_age_ = *1.0) of young children in the United States classified with different states of mind with respect to attachment respond to RS. First, we provide samples of mothers’ AAI discourse to illustrate their states of mind with respect to attachment. Next, we analyze their responses during RS sessions using a qualitative approach. Results demonstrate differences in mothers’ attachment‐related discourse, capacity for reflection, and change processes during the intervention that varied by attachment profile. We discuss the implications of these findings for RS and other parenting interventions.

## 1 INTRODUCTION

Attachment theory posits that children's early experiences in the caregiving relationship set the stage for their social functioning later in life (Bowlby, 1973; Main et al., 1985). Due to their history of receiving sensitive care, secure individuals have accessible, flexible, and internally consistent systems for representing attachment‐related information and developed capacities for mentalizing and self‐regulation (Fonagy & Target, 1997; van der Voort et al., 2014). In contrast, the attachment representations of insecure individuals (dismissing, preoccupied, unresolved)^1^ are characterized by anxiety and/or avoidance and distortions of cognitive processes that focus on threatening attachment content (Dykas & Cassidy, 2011; Mikulincer & Shaver, 2007). Although adaptive in the short‐term, the biases associated with attachment insecurity can impede individuals’ ability to self‐regulate and mentalize about their own and others’ internal states (Fonagy & Target, 1997; van der Voort et al., 2014).

Attachment predicts caregiving behavior across the lifespan (see Bretherton & Munholland, 2008, for a review). For example, parents high in attachment anxiety tend to misread their children's cues and discourage exploration (Selcuk et al., 2010), whereas parents high in avoidance can be less supportive of and responsive to their children (for a review, see Jones et al., 2015). Both forms of attachment insecurity may affect the way parents perceive their parenting experiences as well. Individuals high in attachment anxiety experience heightened anxiety and negative emotion about caregiving when parenting and when away from their children (Gross et al., 2023; Kerr et al., 2021; Mikulincer & Shaver, 2003), whereas avoidant parents experience parenting as less satisfying, less meaningful, and more stressful (Kerr et al., 2019; Rholes et al., 2006).

The Adult Attachment Interview (AAI; George et al., 1996) is considered the gold standard for measuring state of mind with respect to attachment—how individuals represent their childhood attachment experiences. This semi‐structured interview asks interviewees to describe and evaluate their early attachment relationships; verbatim transcripts are coded using a set of scales that capture autobiographical content and the manner in which it is discussed. The scales assess level of coherence (internal consistency; objectivity), metacognition (ability to modify outlook/consider mind of interviewer), idealization (idealization of caregivers), derogation (disparagement/dismissal of caregivers and/or attachment needs), lack of memory (inability/unwillingness to recall attachment content), anger (resentment towards caregivers), and passivity (rambling, off‐topic content) present in the discourse (Main & Goldwyn, 1985–1994). Scores on these continuous “state of mind” scales are used to assign the secure‐autonomous, dismissing, preoccupied, and disorganized attachment classifications. Secure‐autonomous individuals tend to score high on coherence and metacognition and low on the other five scales. Dismissing individuals score high on any combination of the idealization, derogation, and lack of memory scales. Preoccupied individuals tend to score high on the anger and/or passivity scales. Individuals with different states of mind with respect to attachment classifications differ in their capacity for mentalizing (Fonagy & Target, 1997; van der Voort et al., 2014), their valuing of close relationships (Collins & Feeney, 2004), and the degree to which they are dysregulated by attachment‐related content (Bretherton, 2005).

**Key Findings**

1. There is continuity between how participants discuss childhood attachment relationships and their discourse during relational savoring (RS) sessions, particularly during initial RS sessions.
2. Participants with insecure attachment improve in their abilities to engage in child‐focused reflection, remain positive, and be specific over the course of the intervention, while the secure‐autonomous participant demonstrates these qualities from the start.
3. Adapting intervention protocols to the unique attachment needs and sensitivities of participants may optimize engagement with and efficacy of this parenting intervention.

**Statement of relevance**

Relational savoring (RS) is an attachment‐based parenting intervention designed to augment parents’ reflective capacities and improve parent‐child relationship dynamics. This study identifies mothers’ state of mind with respect to attachment as a crucial factor that is associated with engagement with and progress through this promising intervention. Through our qualitative approach, we provide a detailed depiction of how state of mind with respect to attachment can manifest in engagement with RS for parents of young children.

These differences in how those with different profiles of attachment process and reflect upon attachment content manifest in their habitual ways of responding to others (Bretherton & Munholland, 2008; Mikulincer & Shaver, 2003). Given the unique characteristics of each attachment profile, it is critical to evaluate how people with different attachment states of mind react to therapeutic interventions designed to enhance interpersonal relationships, such as the parent‐child relationship, as those with different attachment may require different approaches.

### 1.1 Relational savoring

Savoring is the practice of tuning into, prolonging, and intensifying the thoughts and feelings associated with a positive experience (Bryant & Veroff, 2007; Hurley & Kwon, 2013). Relational savoring (RS) is a specific type of savoring that involves focusing on positive experiences in attachment relationships (Borelli, 2024). When delivered as an intervention, RS involves guiding people to 1) focus on memories of sensitively responding to others’ needs or of others responding sensitively to their needs and 2) enhance the thoughts and feelings associated with those memories (for a description of the protocol, see Borelli, 2024; Borelli et al., 2020). Memories with attachment themes, such as providing or receiving care, acceptance, support, or love from an attachment figure (Bowlby, 1973), are the explicit focus during RS. Whereas general savoring targets the promotion of positive affect and life satisfaction (Jose et al., 2012), RS seeks to augment key interpersonal processes as well (Borelli et al., 2020; Burkhart et al., 2015; Wang et al., 2021).

RS has primarily been examined in the context of parent–child relationships, with parents reflecting on their relationships with their children. In a U.S. randomized trial (*N* = 164), mothers of toddlers who completed four weekly sessions of RS reported greater positive emotion and closeness to their children at post‐intervention, as well as greater parenting sensitivity at the 3‐month follow‐up, compared to mothers who completed four sessions of personal savoring (PS), an intervention focused on individual experiences (Borelli et al., 2022b). Similarly, a group‐based RS study conducted in Iran with mothers of children under age 5 found that, relative to a no‐treatment control, participants in the RS condition reported higher closeness to their children and greater availability at post‐test and at the 2‐month follow‐up (Ansafari et al., 2025). Another Iranian trial of a 4‐week group‐based RS intervention with mothers of children under age 6 replicated these findings, showing that RS was associated with higher maternal closeness, parental satisfaction, and maternal sensitivity/availability at post‐treatment and 3‐month follow‐up compared to a no‐treatment control (Sharifi et al., 2025). Further supporting its efficacy, a laboratory study demonstrated that a single in‐lab RS session, relative to a control condition, increased emotion coaching behaviors among emotionally avoidant mothers (Smiley et al., 2024). Finally, outcomes from a group‐based attachment intervention in which RS was a central component revealed significant increases in both maternal and child attachment security (Borelli et al., 2021). Taken together, evidence supporting RS as an intervention to enhance parenting well‐being and parental behavior is promising and continues to grow.

### 1.2 Attachment and relational savoring

A large body of work has examined the intersection of attachment and psychological interventions (see Levy & Johnson, 2019 for a review). Patient attachment is associated with alliance with the therapist (Diener & Monroe, 2011; Talia et al., 2019), within‐session interpersonal behavior (Talia et al., 2014), and psychotherapy outcomes (Mikulincer et al., 2013).

Associations between states of mind with respect to attachment and client responses to the RS intervention were examined in a recent study: secure attachment predicted higher quality savoring and dismissing attachment predicted lower levels of positivity during savoring (Perzolli et al., 2025). Further, in two studies that assessed attachment with self‐report measures, attachment has been related to trait‐like RS ability (i.e., unguided production of RS narratives). Trait‐like RS ability was measured with the Savoring Quality Scale (SQS; Bond & Borelli, 2017) that codes clients’ memory reflections in terms of detail, positivity, mother‐child emotional connection, and reflection focused on the child. Participants high in self‐reported insecure attachment and low in reflective functioning had lower quality RS responses (Bond & Borelli, 2017; Borelli et al., 2022a), and self‐reported attachment avoidance was associated with lower secure base content (Borelli et al., 2017).

In the current study, we build on this research by qualitatively assessing the ways individuals with different AAI states of mind with respect to attachment respond to the RS intervention. We expect to find linkages between state of mind with respect to attachment and the content of mothers’ memory reflections about caregiving during RS. Moreover, our qualitative approach facilitates a detailed examination of RS narratives that enables us to detect session by session changes in savoring quality. The multiple case study approach has been used by other parenting intervention researchers, for example, to initiate development of a targeted intervention (e.g., with youthful parents who are aging out of the foster care system; Schelbe et al., 2018) or to tailor an intervention to a particular population (e.g., mothers with histories of child sexual abuse; Lange et al., 2020).

### 1.3 Current investigation

This study provides the first qualitative analysis of RS narratives. Specifically, we investigate how *N = *3 mothers with different attachment profiles (one prototypically secure‐autonomous, one prototypically dismissing, one prototypically preoccupied) engage with RS across a four‐session weekly intervention. We chose to examine participants with only primary attachment classifications in this initial illustrative multiple case study design so that we were best positioned to draw connections between state of mind with respect to attachment and intervention response. We start by examining the participants’ responses to two select questions from the AAI, providing excerpts that illustrate how they conceptualize relationships. The central part of the study involves examining how these same participants speak about their relationships with their own children during RS sessions. We apply a qualitative analysis to each participant's RS sessions to generate themes and assess change in savoring quality across sessions. We were particularly attuned to qualities that characterize high quality savoring responses. In other words, we were interested in assessing participants’ ability to generate RS responses that contain high levels of detail, positivity, child‐focused reflection, as well as descriptions of emotional connection (Bond & Borelli, 2017).

## 2 METHOD

### 2.1 Participants

The data used in the present study came from a larger intervention trial (Borelli et al., 2022b). In this trial, English‐proficient mothers (*N = *164; *M_age_* = 30.8 years, *SD_age_ = *5.0) with at least one child between the ages of 18 and 27 months (*M_age_* = 20.9 months, *SD_age_ = *2.4) were recruited to participate in a randomized trial of RS. Study procedures were approved by institutional review board prior to data collection (Pomona College IRB, #4/ 29/2016JB‐MP). One hundred and thirty‐four mothers and their toddlers completed the intervention and the three‐month follow‐up assessment. Families were recruited via online advertisements (e.g., Internet classifieds, social networking sites) and flyers in the community. Children diagnosed with a developmental disability or delay was excluded. All participants provided informed consent prior to taking part in the study.

For the current study, we selected participants (*n = *3; *M_age_ = *31.0 years, *SD_age_ = *1.0) who had participated in the RS condition and whose AAIs were coded as prototypically secure, dismissing, and preoccupied. To select participants, we first identified those mothers in the sample who were assigned exclusively a primary attachment classification. (It is not unusual for AAI transcripts to be given a secondary attachment classification if they represent attachment‐related information reflective of two or more different classifications.) This initial screen yielded nine participants (four autonomous, three dismissing, two preoccupied). Using an online number generator, we then randomly selected one participant from each attachment classification to be included in the current study. Two of the three mothers identified as White, non‐Hispanic/Latina and one mother identified as Hispanic/Latina. All participants reported having obtained a bachelor's degree. The target children (*M_age_ = *20.0 months*, SD_age_ = *1.73) included two females and one male. All case material presented from these participants is de‐identified and anonymized.

### 2.2 Measures

#### 2.2.1 Adult attachment interview (AAI)

The Adult Attachment Interview (AAI; George et al., 1996) is a widely used measure of state of mind with respect to attachment. To generate adult attachment classifications, participants’ narratives are scored quantitatively by certified coders employing the AAI coding protocol (Main & Goldwyn, 1985–1994). Coders use the state of mind with respect to attachment scales (i.e., coherence, metacognition, idealization, derogation, lack of memory, anger, passivity, plus scales for unresolved loss or other trauma that are not included in this report) to assess and classify state of mind with respect to attachment. AAIs from our full sample were coded by a certified coder and 21% of cases were coded by a second certified coder, with acceptable interrater reliability on overall classification (**Κ** on 4‐way classification = .64, **Κ** on 3‐way organized classification = .62) and coherence (*ICC* = .79).

#### 2.2.2 Savoring quality scale (SQS)

The Savoring Quality Scale (SQS; Bond & Borelli, 2017) assesses the quality of participants’ responses to RS prompts. On a 5‐point Likert scale, coders rate the extent to which responses included: 1) a *specific*, *positive* memory between the mother and child; 2) aspects of *emotional connection* between mother and child; and 3) *child‐focused reflection*. While previous research has utilized the SQS scores to quantify RS quality within‐ and between‐sessions and demonstrated excellent reliability (*ICC* = .95; Bond & Borelli, 2017), the SQS can also be employed qualitatively. Specifically, the three SQS dimensions (specificity and positivity; emotional connection; child‐focused reflection) can inform qualitative approaches for generating themes. In the current study, the SQS was only used qualitatively to guide the analysis of RS discourse, as described below.

### 2.3 Procedure

As part of the larger study from which the present data were drawn (Borelli et al., 2022b), mothers came to the lab for a baseline assessment during which they completed the AAI. Next, mothers were randomized to condition (RS or PS). Savoring sessions were administered in home once per week for four consecutive weeks. In RS sessions, trained paraprofessional interveners led mothers through a minute‐long mindfulness breathing exercise, followed by the two phases of RS: memory selection and memory reflection (see Borelli, 2024 for more information). In memory selection, mothers were guided to generate several memories of positive connection with their toddler. After the intervener determined which memory had the most salient attachment content (secure base, safe haven, close connection), vivid detail, positive emotion, as well as the least potential for spoiling by negative emotion, they began memory reflection. Interveners helped participants to deeply reflect upon the selected memory by posing prompts or questions about the sensory details, emotions, and meaning associated with the memory. At the end of the reflection period, parents were asked if they had any further thoughts about their experience. Savoring sessions were audio‐recorded and lasted approximately 15–40 min.

#### 2.3.1 Qualitative coding method

We first examined participants’ AAI discourse in response to two questions that tend to evoke salient attachment content: (1*) Are there any aspects of your childhood you would consider a setback?* and (2) *Why do you think your parents behaved the way they did during your childhood?* Our goal was descriptive: to illustrate the ways that participants represented their own attachment relationships. For each participant, we highlight narrative elements that speak to the state of mind scales (e.g., coherence, lack of memory) as well as elements linked to attachment in the research literature—namely, emotion regulation (Bretherton, 2005), valuation of relationships (Collins & Feeney, 2004), and mentalizing capacity (van der Voort et al., 2014).

Second, to analyze participants’ verbal responses during the RS sessions, we employed thematic analysis (TA; Braun & Clarke, 2012). TA is a flexible method for identifying, organizing, and offering insight into patterns of meaning across long‐form linguistic data. With TA, coders initially read a transcript to generate themes—i.e., brief summaries of sections of the transcript. After generating and synthesizing themes from the transcript, coders conferenced the themes. This step allows coders to adjust, remove, add, and eventually agree upon the qualitative themes in the transcript. Depending on the nature of the data and the focus of the research, TA can involve an inductive, bottom‐up approach driven largely by what is found in the narrative and/or a deductive, top‐down approach in which the researcher adopts a particular theoretical lens to interpret and code the transcript.

For the RS analyses, we employed largely a top‐down approach, such that our coding was guided by the three SQS dimensions of savoring quality (specificity and positivity; emotional connection; child‐focused reflection). However, we also attended to emergent details of the narratives that were not necessarily tied to the SQS scales (bottom‐up approach). First, two coders independently read the secure participant's first RS session. During this initial read, each coder wrote brief summaries of the participant's responses to the five RS prompts within that session. In these summaries, coders noted the participant's descriptions of emotional connection and their capacity to be specific, positive, and child‐focused (dimensions of the SQS) but did not yet generate themes. Next, each coder independently reviewed their summaries of each RS prompt and identified themes that were present in multiple responses, resulting in one set of themes for that first RS session. They repeated this process for the three remaining RS sessions. After each coder had reviewed and generated a preliminary set of themes for all four RS sessions, they met to review themes. During these meetings, each coder presented the themes they identified for each RS session, the rationale for each theme, and the excerpt(s) from which the theme was drawn. Themes identified by both coders that were nearly identical in content were retained. Disagreement, defined as occurring when a theme was identified by only one coder, was resolved through discussion until 100% agreement was met. The consensus process resulted in a final set of themes and their respective, illustrative excerpts that had been mutually agreed upon by the coders. Excerpts presented in Results capture the consensus themes. This same process was followed for the dismissing and preoccupied participants.

### 2.4 Positionality and reflexivity

The TA was conducted by the first author and the third author. The first author identifies as a white, cisgender man with no experience as a parent. He is currently a doctoral student in clinical psychology, with training in relational psychodynamic therapy. Since 2016, he has been involved with research that adopts an attachment framework and is trained to administer a number of attachment interviews as well as the RS intervention. He is certified to code the Working Model of the Child Interview (WMCI: Zeanah et al., 1996).

The third author identifies as a biracial, cisgender woman with no experience as a parent. She has been trained in the RS intervention. She has conducted attachment‐based research since 2018 and is currently a doctoral student in clinical psychology, with training in mentalization‐based treatment (Bateman & Fonagy, 2009). She is also certified to code parental reflective functioning from the Parent Development Interview (Slade et al., 2004).

The coders’ research backgrounds make them particularly suited to code narrative data for attachment themes. However, their training and the fact that they are not parents also presents potential sources of bias that was acknowledged throughout the coding process. They frequently engaged in reflexivity, assessing how their background, experiences, and knowledge might influence their interpretations. On several occasions, each of the coders sought feedback from other study authors (all of whom are parents) to get feedback on how they were thinking about a general aspect of parenting that came up during coding. To facilitate transparency and provide the reader with access to participants’ narrative data in their own words, verbatim excerpts from which the themes were drawn are provided.

## 3 RESULTS

### 3.1 AAI discourse and RS quality of the secure mother

#### 3.1.1 Examples of attachment discourse during the AAI

Below we illustrate evidence of coherence, metacognition, emotion regulation, high valuation of relationships, and mentalizing.

When asked if aspects of her early experiences felt like setbacks, she states:

*I was diagnosed with ADHD when I was like 8 or 9 um and so the way that manifested I was in trouble a lot at school…I understood the information, but so much of my school day was about my weaknesses sitting still, being quiet, um being in order, um, as opposed to, um, finding a way to meet, meet me where I was at and allow me to grow…But I think had those things been done differently, I think I would have been able to thrive in life sooner than I was able to. Does that make sense?*

Here, the participant displays her ability to coherently evaluate her childhood experiences from an objective, adult perspective. She is not emotionally threatened by considering how some aspects of her early life might have held back her development. Asking the interviewer at the end if she needs to clarify her answer (“Does that make sense?”) may be evidence of a metacognitive capacity to consider the mind of the interviewer.

When asked why her parents behaved as they did during her childhood, she responds:

*I think that as I've been kind of able to see my parents’ backgrounds differently like from an adult perspective as opposed to a child, the way they behave makes a lot more sense to me. And that for me was really, really freeing…It was, it was all about like kids are seen and not heard, and I just didn't fit that. I mean just very precocious and I never felt the need to call adults by their last name…They were kind of like, “What the heck do we do with this girl, who just, who won't simply comply?” Um, and so I think they were having to write a new rule book. And it's just, it's interesting*.

The participant's developed mentalizing capacity seems to allow her to recognize her parents’ as well as her own contributions to the caregiving dynamic. She presents a balanced perspective regarding her personality and family norms, while remaining sympathetic to her parents. At the end of the excerpt, her interest in thinking about these dynamics indicates openness. Her sophisticated mentalizing capacity allows her to draw personally meaningful insights regarding her childhood attachment relationships.

#### 3.1.2 RS quality

In the section on RS quality, for this participant as well as for the other two, we underline identified themes.

In the first home visit, the participant reflects upon a memory of being at the beach with her child. When the intervener asks what she was thinking at the time, she responds:

*I was thinking that we were really close to each other, um, and that how by next summer, she probably isn't going to need the same level of closeness. Um, that she'll be more ready to kind of just run off on her own…So, I was trying to really savor and enjoy how much she wanted to be held and how much she needed me right then*.

The participant focuses on her connection with her child and engages in child‐focused reflection, describing their emotional and physical closeness (emotional connection) in the moment but also her daughter's need for independence over time. Considering a child's need for more independence often causes parents to veer towards negative thoughts and feelings; indeed, the RS manual specifies strategies for addressing so‐called “spoiling” (Borelli et al., 2020). This participant, however, remains highly positive, describing how she is driven to “really savor and enjoy” her child expressing her needs (valuing of relationships). (Note that participants were not told the name of the intervention, and the words “savor”/“savoring” are not mentioned in the instructions to participants nor in the consent or recruitment materials.)

At the end of the first home visit, the intervener asks the participant to let her mind wander, encouraging her to express any new thoughts she has. The participant mentions:

*“I was also noticing, like, how she really wants to please me. And, like, you know, I could see dolphins, and she doesn't yet like get what a dolphin is ‘cause she hasn't closely encountered one yet. And you know we've seen them in books… It's not something that's right in front of her vision so you know. I'm like trying to show her where the dolphin is, and she's like looking but you know her eyes aren't going to where I'm pointing and she's like, “Yeah there it is! It pops up.” And so, it's just kind of funny that like she is trying to, to just be like, “Mom says it's there, I'm going with it.” You know, just that her trust is, is there. So that was really sweet.”*

The participant generates a highly specific, positive, and child‐focused, memory, meeting all three criteria of SQS. Rather than viewing her daughter's behavior as simple behavioral imitation, this mother perceives her daughter's epistemic trust and appreciates being relied upon (sophisticated child‐focused reflection). Further, this mother's discourse seems to convey a sense of delight in connecting with her daughter, a central component of the secure attachment relationship (Britner et al., 2005).

During the third home visit, she recalls being in the pool with her child:

*I think a couple weeks ago we were swimming, and, I um, I think I let her, let her face dip under the water a few times which made her nervous. So, this time she was really hesitant at first. So, I was trying to, um, create some different ways to make it fun and to help her float…And at first, she struggled and fought, but when she could feel my hands under her head she eventually—I'd say, “Relax your arms,” and she would, and she would feel the float— she'd feel herself floating. And she'd like smile and then realize she was doing it and then like struggle. So, it was just kind of funny. Like, she liked it but was scared but then would relax but then— So, it was just kind of fun to see the process for her*.

In this excerpt, the participant helps her child through an anxiety‐provoking experience. She considers the mind of her daughter, reasoning that her previous experience made her hesitant. At the same time, watching her daughter's behaviors and emotional reactions is enjoyable (delight). Although the memory contains some negativity (i.e., her child's anxiety), the takeaway of the memory is highly positive and brimming with child‐focused reflection.

In sum, from the first session, this participant embraced RS; she did not need much prompting and seemed to enjoy the process (high quality engagement at baseline). She readily produced highly specific, positive memories; gravitated towards moments of emotional connection; demonstrated an inquisitive stance towards her child's internal; and exuded a sense of delight in her relationship with her child.

The quality of her savoring resonates with the narrative elements of her AAI. In the AAI, she comfortably mentalized about underlying causes of the dynamics between her and her parents, and while savoring, she mentalized about her child's internal states. Her overall comfort discussing close relationships in the AAI is mirrored in her meaningful engagement with RS. Just as in her AAI, when she was regulated emotionally while discussing her recollections, during RS she was regulated and positive despite musing about her daughter growing more independent. Similarly, she was able to acknowledge and reflect upon her child's anxiety without becoming mired in the negative. More broadly, both the AAI and the RS transcripts are marked by their overall coherence. The comparison of the two narratives suggests continuity in the representations of her childhood attachment relationships and her current relationship with her own child.

### 3.2 AAI discourse and RS quality of the dismissing mother

#### 3.2.1 Examples of attachment discourse during the AAI

Below, we illustrate evidence of lack of memory, idealization, deactivation of emotion, low valuation of relationships, and rudimentary mentalizing.

When asked if there were any aspects of her early experiences that held back her development, this participant responds:

*“No”*

When the question was rephrased and asked again, the same response was given. This mother might believe that nothing in her childhood held her back or she might be unable or unwilling to reflect upon negative childhood experiences. For dismissing participants, negative emotionality can feel threatening. Insisting that negative elements of her upbringing—which every child is likely to experience—were absent may be a strategy to deactivate and reduce feelings of vulnerability.

Towards the end of the interview, the participant is asked why she thinks her parents behaved the way they did during her childhood. She responds:

*I mean, I guess that's just their personality. Maybe they just learned that from others too*.

Rather than providing specific reasons for her parents’ behavior, such as their emotions, goals, or values, she links her parents’ behavior to the broad concept of “personality” and to the notion that they learned “from others.” Her capacity to reflect upon her childhood attachment relationships seems limited. Employing curt, behaviorally focused answers in response to attachment‐related questions is an example of deactivation (Edelstein & Shaver, 2004). With a tendency to deemphasize close relationships and attachment‐related information more generally, this participant may lack the motivation or ability to reflect on the nuanced dynamics that characterize interpersonal relationships.

#### 3.2.2 RS quality

In the first visit, the participant savors a memory of a time when she was doing laundry and her child was helping to pick up the clothes. When asked about her thoughts, she responds:

*So, like, I was, I didn't really think about it too much, but you know, it was kind of like a bonding experience where we're able to do, like, housework together. So, you know, I'm proud to be her parent, and I'm happy she's able to feel like she's helping me*.

In this excerpt, the dismissing mother recounts her daughter doing something alongside her and calls it “a bonding experience.” However, the description of the episode lacks detail and the joy of close connection seems missing (low emotional connection). Nevertheless, she remains focused on a moment of connection between her and her daughter. The attachment language she uses (“bonding”) and her openness to engaging with this process in the first RS session is noteworthy and somewhat unexpected given her dismissing attachment classification.

Later during the reflection, when asked how their bond will affect their relationship in the future, she says:

*I'd say when she's a teenager, I know we realistically probably won't get along…. And I guess as she gets older that we would have more of kind of like a friendship, but I guess, like I try to be more strict with her now because I want her, you know just the way the world is going, like I want her to be a good person. I also want her to be independent in a way where you know, she'll still need me, but she'll be able to handle things on her own*.

Here the mother begins by expressing a pessimistic view for the future relationship with her child (low positivity). Of note, the question asks about the development of the caregiving relationship, but her response does not mention thoughts and feelings of her child (low child‐focused reflection). Rather, she focuses on her current parenting behavior and on broad personal qualities she hopes her daughter will acquire (being a “good person,” “independent”).

In the second home visit, she savors a memory from earlier that day:

*I saw that she was kind of happy and kind of quickly running to me. Like, I knew instead of just brushing it off and saying “Oh, hey baby,” I tried being more animated and show her I was happy to see her too. I was kind of proud of myself to do that because I'm not the type that's animated or show their love kind of type. So, I'm really trying to work on that with her*.

This excerpt is strikingly different from the previous two. It contains a greater degree of positivity and specificity. She recounts a positive interaction with her daughter and engages in more sophisticated child‐focused reflection (as well as self‐focused reflection). Further, she describes consciously augmenting her positive emotion (“I tried being more animated,” “I'm really trying to work on that”) and savors the feeling of being “proud” of herself for making this adjustment for her daughter. After just one savoring session, this mother seems to have a stronger capacity to remain focused on positive emotional content.

In the last home visit, she reflects on their relationship in the future:

*Like throughout this whole process I've just kind of be like more aware and notice little things like that you know [child] may need me to comfort her, to like protect her, to reassure her. So like every day I try to take it day by day. Little by little so that, you know, hopefully, not just in the future when she's older, but like tomorrow, like, if she were to fall, or needs me, or something then, you know, she'll know that, “Oh, mom's going to come when I cry.” So I think she's securely bonded to me but, you know, I hope that as she gets older, that she'll realize that I'm there for her when she needs me*.

In the beginning of this excerpt, this mother describes some of the ways engaging in RS has positively affected her, using child‐focused, attachment‐based language (emotional connection, child‐focused reflection). In this excerpt as well as in the previous one, she reflects on how she responds to her child and describes her efforts to be present for her child. Further, her optimism regarding their future relationship has grown (positivity). She now expresses hope that as her daughter ages, she will understand that she can always turn to her for support.

This participant encountered a few areas of difficulty during the intervention. Especially initially, her responses to RS prompts were short, behaviorally focused statements. Further, she used simple emotion labels and descriptions of mental states. These tendencies are consistent with her AAI discourse, where she deflected questions that asked about negative experiences and attachment relationships. It is noteworthy that emotion deactivation occurred when prompted to reflect both on negative experiences (in the AAI) and positive experiences (during RS). Through repeatedly engaging with RS, however, she seemed to strengthen her capacity to focus on specific, positive moments, on how her caregiving responses are perceived by her child, and on emotional themes. These changes suggest that this mother may be learning to respond to the attachment needs of her child in a different manner.

### 3.3 AAI discourse and RS quality of the preoccupied mother

#### 3.3.1 Examples of attachment discourse during the AAI

Below we illustrate evidence of passivity and/or anger, emotion dysregulation, high valuation of relationships, and rudimentary mentalizing.

When asked if she felt any aspects of her early experiences were a setback, this participant responds:

*I was hit with constant negativity. It's really hard to think that you're okay at something if you're never good enough. Um, as simple as sweeping the floor, to this day if you ask my dad, I still can't sweep the floor right…. Um, but you were never good enough, you were always doing it wrong…. So, you know, that's hard to understand. Um, and then you just get past it, and you are who you are, and you are going to deal with it, and I can talk to you, or I can't talk to you*.

In this excerpt, the participant has difficulty confining her narrative to her childhood experience, mentioning that she still cannot do certain things correctly according to her dad. She delivers an emotionally fueled response that contains vague language, distancing pronouns (i.e., “you”), and is hard to follow. She exudes active frustration describing her childhood narrative.

When asked why her parents behaved as they did during her childhood, she responds:

*She was as good as I think she knew how to be, um, given what she grew up with. And I didn't know my grandmother was an alcoholic until many years later…. So, I knew she was an alcoholic when my mom was a little girl, and I imagine that's tough to deal with. Um, my brother‐in‐law is actually six months sober now and we've been dealing with him, trying to tell him forever he has an issue…. Um, and my dad didn't, his parents weren't around, so he wasn't around. But it was okay, um, not okay. But I think that's the way they knew that that is what they had ingrained in them, how to deal, how to cope. That's what you do with the kid—let it sit there*.

Here, this participant draws connections between her mother's childhood and how she was parented; however, rather than mentalizing about how being cared for by a mother struggling with alcoholism might impact her mother's ability to parent, she shifts to the unrelated topic of her brother‐in‐law's sobriety. When she mentions her father, she follows a similar discourse pattern, using vague, rambling sentences and contradictory statements (“it was okay, um, not okay”). By the end, it is unclear to whom she is referring. Her discourse is disjointed and hard to follow, demonstrating an anxious orientation towards her attachment history and difficulty mentalizing in a regulated manner.

#### 3.3.2 RS quality

In the first session, the participant focuses on a memory of giving her son a bath. Describing how their bond will affect their future relationship, she says:

*I hope it paves the way for open communication if anything, that we continue to go over body parts together in the bathtub, and brush our teeth…And, but oh my goodness, I hope that he knows that we are always there to try to teach him something. And that we'll help him in any way we can, so I would love that. I know at some point he is going to hate us too, so I am not ready for that one*.

In the beginning of this excerpt, this mother conveys an openness to discussing attachment themes, touching upon her hope that her son views her as a secure base (“I hope that he knows that we are always there to try to teach him something”). She seems to value themes that are central to secure attachment, such as trust, communication, and support provision and perspective takes for the child (valuing of relationships, child‐focused reflection). Towards the end of the excerpt, however, she veers negative (low positivity). Even as she expresses hope for her future relationship with her child, she simultaneously voices anxiety about the inevitable deterioration of their relationship (interpersonal anxiety).

During the second home visit, she describes a memory of a time when rather than going to her husband's lap before bed, her child came to her:

*Yeah, it's nice to feel that, you know, you want to consciously wanted to be there, not be rejected as soon as someone else better comes along or, you know, good cop because mom is bad cop. And yeah daddy doesn't put me in time out, but mommy does. I am going to go over here, so it was very nice just to be wanted and that's, you know*.

Generally, this response shows a lack of focus on the child and the dyadic nature of the interaction. Although attempting to describe a moment of connection with her son, this mother is self‐focused and negative, mentioning how she is usually rejected by her child “as soon as someone better comes along” (low child‐focused reflection, low positivity). Here the experience of being wanted (or not) may automatically elicit anxiety and other negative thoughts/feelings (interpersonal anxiety). Additionally, the way this mother switches between first‐, second‐, and third‐person pronouns makes the response difficult to follow (lower coherence).

In the third home visit, this participant savors a memory she has of reading a book about colors with her child:

*I can't wait to see where it goes next week. Honestly, I can hope that next week he identifies orange or yellow or brown…. But he definitely took some likes in primary colors which is neat. Yeah, I just hope he continues. Maybe you know he will start counting or something … So, I can't wait to see where we, he goes, you know, tomorrow even*.

The reader gets a sense here that this parent is genuinely excited about engaging with her child through reading, in comparison to the anxiety that pervades much of her discourse. The positivity of this excerpt, particularly how it ends (“I can't wait to see where… he goes… tomorrow even”), is in stark contrast to the discourse from other sessions in which positive themes are quickly followed up with anxiety or pessimistic statements regarding their relationship. It may be a sign of growth that this mother remains positive and specific here.

During the fourth home visit, the participant savors comforting her child:

***“*** *Since the beginning he's been able to come to us, which is nice cause I couldn't feel like I could always go to my parents…. I would say definitely like around five you couldn't speak to them. Um, but it's nice. It's not to say it's not annoying sometimes, um, but it's good. Trying [laughter] hopefully trying. I feel like I'm failing most of the time, but I'm still trying.”*

In this excerpt, reflecting upon a time when her child came to her after getting hurt (emotional connection), she compares her own experiences being parented with the experiences of her child to highlight discrepancies in parental responsiveness. Notably, she evaluates a negative aspect of her upbringing from an adult perspective and describes trying to adjust her caregiving for her child's benefit (child‐focused reflection). The controlled manner in which she speaks about her childhood here contrasts with previous descriptions that were riddled with lingering feelings of anger. RS may have allowed her to reflect upon her attachment history with a higher degree of objectivity and acceptance. Finally, while she expresses self‐doubt about her parenting at the end, she also recognizes her efforts and seems to be invested in the process of growth (“Trying… I'm still trying”).

Overall, this mother's RS transcripts were considerably longer than the others. She sometimes mentioned unrelated details, spoke in run‐on sentences, and alternately used first‐ and second‐person pronouns for herself. Further, particularly early on, interpersonal anxiety permeated much of her RS discourse. These qualities, which are paralleled in the rambling nature of discourse on the AAI, contribute to the general lack of specificity and clarity of her RS responses. However, over time she increased her ability to remain focused on positive relational topics, particularly in the final two excerpts in which she expressed a sense of enthusiasm and hope. That said, her tendency to veer towards the negative when discussing attachment content remains. During the fourth session, she generalizes in a positive way about her relationship with her child, commenting that he has always been able to come to her for support, which is different from how she felt about her own parents. Although elements of preoccupation are still present, she clearly has strengthened her capacity to reflect and remain focused upon specific moments of emotional connection with her son.

## 4 CONCLUSION

Participants’ attachment profiles are associated with how they perceive and respond to interventions (Levy & Johnson, 2019), and this may be especially true for interventions that are relationally focused. Through our analyses, we shed light on the associations between state of mind with respect to attachment and RS intervention responses. The secure participant produced high quality RS responses from the first session and the two insecure participants demonstrated unique areas of difficulty and trajectories of change. Below we summarize and compare fundamental characteristics of the three participants’ AAI discourse that re‐emerged in the quality of their savoring responses.

The AAI discourse of the secure participant was characterized by coherence, sophisticated mentalizing, metacognition, valuing of close relationships, and capacity for emotion regulation. During RS, the participant's capacity to discuss relational content helped her to identify poignant moments of connection with ease and explore her daughter's internal experience with delight. Overall, for this participant, RS seemed to resonate with her strong reflective tendencies. She clearly found the RS intervention satisfying and, given her enthusiasm for it, a practice that she might incorporate into her parenting toolbox.

In comparison, the dismissing participant was generally unwilling or unable to reflect upon attachment themes during the AAI, exhibiting rudimentary mentalizing and avoiding discussion of negative experiences. However, during the first two sessions of RS, her responses conveyed an openness to engaging in RS that was somewhat surprising given her AAI discourse. It is possible that discussion of her relationship with her child does not trigger deactivation and avoidance to the same extent that discussion of her childhood attachment relationships does. However, like her AAI, these early RS sessions were behaviorally focused, lacked detail, and contained minimal child‐focused reflection. Excerpts from her last two sessions, however, contained specific descriptions of emotional connection and positivity even though they were not highly nuanced. Using relationally oriented words (i.e., “comfort,” “protect,” “reassure,” “securely bonded”), she described her child's inner experience as well as her efforts to modulate her own emotions for the benefit of her child. In the final excerpt, she noticed a shift in the way she perceives her child's internal states, seeing more and more “little things” and prioritizing attuned caregiving responses. This shift mirrors empirical research in which dismissing participants are particularly touched by savoring, showing the most improvement in caregiving sensitivity, positive emotion, and relationship satisfaction (Burkhart et al., 2015; Smiley et al., 2024).

In contrast to the emotional deactivation of the dismissing participant on the AAI, the preoccupied participant showed heightened anxious emotion. Similarly, during RS, even though the mother was prompted to focus on positive aspects of her memory, to varying degrees, each response contained an element of rejection, worry, annoyance, or fear. For those high in attachment anxiety, an instruction to ignore or suppress negative emotion can instead increase negative and decrease positive emotion (Ben‐Naim et al., 2013). Although her responses to RS suggest that she is psychologically oriented toward attachment, as is typical for those with higher attachment anxiety (Mikulincer & Shaver, 2003), close relationships (and reflecting on close relationships) tend to evoke anxiety. In later sessions, while a negativity bias remains, her ability to remain positive seemed to improve and she began to mentalize for herself and her child with more sophistication.

A common theme emerged for all three participants—they each mentioned that their relationship with their child would inevitably change—but their descriptions of change and the feelings it evoked were quite different. The dismissing participant frankly stated that their relationship would likely deteriorate (“When she's a teenager, I know we realistically probably won't get along”). In contrast, the preoccupied participant anticipated inevitable relationship struggles (“I know at some point he is going to hate us too…I am not ready for that one”). The secure mother stated that she was motivated by the notion of diminished closeness in the future to “really savor and enjoy” how much her child needs her in the moment. Each participant's comments reflected their state of mind with respect to attachment: whereas the dismissing mother immediately seized upon a negative theme, the preoccupied mother became overly involved with it, and the secure participant acknowledged the possibility of decreasing closeness but also derived in‐the‐moment meaning and positivity from it.

### 4.1 Clinical implications

One of the core goals of RS is to help parents focus upon and develop meaning from the small moment‐to‐moment positive caregiving experiences that oftentimes are unnoticed. As our analysis has shown, even at baseline, the secure participant operated within a relationally oriented framework and naturally focused on salient moments of connection with her child to develop meaning. This pattern has at least two important implications for secure participants: they may benefit from brief (i.e., single session) courses of RS because it comes “naturally” to them, and they might require less intervener guidance to benefit from the intervention (e.g., a group therapy‐based approach to RS [Borelli et al., 2021], prompts from a digital interface).

The two insecure participants encountered more difficulty during RS and, especially initially, produced lower quality RS responses. To more effectively engage parents or clients with insecure attachment styles in RS, pre‐screening for attachment style could help interveners target areas of specific weakness, including parents’ baseline capacities to remain positive and to engage in nuanced reflection, as these appear to be critical factors for optimal engagement.

With dismissing parents, interveners could help them cultivate emotion and positivity in their reflections. Before an RS session, security priming—experimentally enhancing felt security, comfort, and safety with words or images—is one approach that might help dismissing participants remain positive during RS (Gillath & Karantzas, 2019). During RS, interveners could prioritize verbal and nonverbal exploration of affect using music or photos of specific remembered events (Quoidback et al., 2015). In comparison, preoccupied individuals might benefit when interveners help them create some distance between their own childhood memories and their current caregiving relationships to reduce dysregulation and relationship anxiety. For example, a key Dialectical Behavioral Therapy (DBT) distress tolerance skill known as “Push Away” helps clients contain negative affect by temporarily setting aside the source of distress through mental imagery (i.e., putting the problem in a box on the shelf; Linehan, 1993). It is possible that such an approach might help anxious individuals to temporarily tamp down negative emotion.

For both dismissing and preoccupied mothers, our session by session analyses suggest that they each benefitted from intervener support for RS over time. That is, intentionally drawing attention to attachment themes at each of the four sessions appears to enable them to appreciate their caregiving roles and to experience more positive emotion when reflecting on caregiving interactions. Indeed, preliminary findings suggest that intervener fidelity to the principles of RS (redirecting attention to the positive, calmly matching participants’ tone, eliciting secure base content) predicts key outcomes of the intervention, such as participant reflective functioning and closeness to child (Borelli et al., 2024a). Intervener efficacy could be enhanced by viewing videos of RS sessions of individuals with different profiles of attachment and teaching intervention techniques for addressing prototypical challenges that we highlight above.

### 4.2 Limitations and future directions

This study is the first to qualitatively examine thematic shifts in RS engagement over the course of a month‐long intervention through the lens of participants’ attachment states of mind. Despite the links we uncovered, there are certain limitations to our analysis. First, the similar education levels of the three participants (each earned a B.A.) limits generalizability, as metacognitive constructs (e.g., reflective functioning) are associated with sociodemographic factors such as education (Sleed et al., 2020). Additionally, in this initial illustrative multiple case study, we analyzed data from participants with only primary attachment classifications to illustrate how RS discourse can vary according to state of mind with respect to attachment. However, many participants are also assigned secondary attachment classifications because their discourse patterns are associated with more than one attachment classification. For instance, a participant might describe their childhood attachment figures in a detailed and objective manner but also report that they experienced no setbacks in their childhood. A participant with these narrative elements may receive a secure primary attachment classification and a dismissing secondary attachment classification, or vice versa, depending on which attachment strategy appeared dominant. Our approach of selecting participants with only a primary attachment classification, while intentional, limits the transferability of study findings. Findings from the present work should be interpreted with caution because, in general, participants in an RS intervention are likely to display multiple attachment strategies at different times.

Further, RS narrative excerpts were not randomly chosen. Rather, excerpts were selected based on their adherence to themes identified through TA; this method limits internal validity. Including additional quantitative outcome measures, in a study with a greater number of participants, would strengthen our conclusions about the ties between mothers’ attachment states of mind and their engagement with RS, and thereby, the external validity of the research. Additionally, the fact that coders were aware of the attachment classifications of each participant is a limitation, as this knowledge may have biased their coding of the RS sessions. Future work in which coders are blind to participants’ states of mind with respect to attachment is merited. Finally, we did not assess psychopathology or other variables that could impact mothers’ engagement with RS.

Despite these limitations, our findings have clinical implications for researchers developing parenting interventions and for clinicians working with parents. Specifically, our findings suggest that it may be important to tailor treatment protocols to target specific areas of attachment‐related vulnerability. In recent work, parenting interventions have been tailored to the *cultural* backgrounds of families; these have yielded positive outcomes for both parents (Borelli et al., 2024a; Holtrop et al., 2023; Parra‐Cardona et al., 2023) and youth (Borelli et al., 2024b). Adaptations of parenting interventions have also been explored for alternate caregivers (e.g., grandparents) by qualitatively assessing their needs (Kirby et al., 2012). Study findings provide preliminary support for the idea that tailoring the RS intervention to participants’ attachment profiles may be beneficial. However, further work is needed to support such adaptations.

Future research could examine the hypotheses and findings from the present work quantitatively. One extension of this initial illustrative multiple case study design might involve quantitative coding of attachment‐related discourse during RS, which would allow researchers to assess coder reliability and quantify change over time. Quantitative methodological approaches with larger samples would also allow researchers to test the magnitude of the differences in participants' capacity to be child‐focused, positive, and specific during RS based upon their state of mind with respect to attachment. Mixed methods approaches that involve triangulation of qualitative and quantitative data may be optimal for study questions involving intervention adaptation. Such an approach could improve generalizability while retaining interpretative richness.

Future work might also include individuals with blended attachment classifications (e.g., combined dismissing, preoccupied). One way to approach this research question would be to measure attachment dimensionally, taking account of participants’ levels of attachment avoidance and attachment anxiety (e.g., by using Attachment Style Questionnaire; Feeney et al., 1994). Then, using a variable‐centered approach, researchers could test associations between attachment avoidance and anxiety and engagement with RS. Moreover, it could be fruitful to examine how participants with different states of mind with respect to attachment engage in other attachment‐based interventions, such as the Circle of Security and TheraPlay (Booth & Jernberg, 2009; Powell et al., 2013). This work could identify broader patterns of how those with different states of mind with respect to attachment engage with and respond to attachment‐based interventions.

## CONFLICT OF INTEREST STATEMENT

The authors declare no conflicts of interest.

## FUNDING INFORMATION

Funding for this project was awarded by Pomona College Faculty Research Grants (Jessica L. Borelli and Patricia A. Smiley), David L. Hirsch III and Susan H. Hirsch Research Initiation Grant (Jessica L. Borelli), and the American Psychoanalytic Association Grant (Jessica L. Borelli).

## Data Availability

The data that support the findings of this study are available from the corresponding author upon reasonable request.

## ENDNOTE

1 Note that the terms for different attachment orientations vary as a function of the measurement tool used. Avoidance, anxiety, and security derive from the social psychological tradition of measuring attachment styles via self‐report measures, whereas dismissing, preoccupied, and secure‐autonomous attachment derive from the developmental/clinical tradition of measuring attachment using interview or behavioral observation measures. In the current paper, when describing studies, we use the terms that match the measurement modality employed. When describing principles that apply to both measurement traditions, we use both terms (e.g., dismissing/avoidant). Although these different measurement traditions use different terms to describe attachment, they have the same theoretical roots, even though they are typically not strongly correlated with one another (see Roisman et al., 2007, for a review).
